# Temozolomide Resistance in Glioblastoma by NRF2: Protecting the Evil

**DOI:** 10.3390/biomedicines11041081

**Published:** 2023-04-03

**Authors:** Karoline Almeida Lima, Isabeli Yumi Araújo Osawa, Maria Carolina Clares Ramalho, Izadora de Souza, Camila Banca Guedes, Cláudio Henrique Dahne de Souza Filho, Linda Karolynne Seregni Monteiro, Marcela Teatin Latancia, Clarissa Ribeiro Reily Rocha

**Affiliations:** 1 Department of Clinical and Experimental Oncology, Federal University of Sao Paulo (UNIFESP), Sao Paulo 04037-003, Brazil; karoline.almeida@unifesp.br (K.A.L.);; 2 Laboratory of Genomic Integrity, National Institute of Child Health and Human Development, National Institutes of Health, Bethesda, MD 20892-3371, USA

**Keywords:** NRF2, glioblastoma, temozolomide

## Abstract

The transcription factor NRF2 is constitutively active in glioblastoma, a highly aggressive brain tumor subtype with poor prognosis. Temozolomide (TMZ) is the primary chemotherapeutic agent for this type of tumor treatment, but resistance to this drug is often observed. This review highlights the research that is demonstrating how NRF2 hyperactivation creates an environment that favors the survival of malignant cells and protects against oxidative stress and TMZ. Mechanistically, NRF2 increases drug detoxification, autophagy, DNA repair, and decreases drug accumulation and apoptotic signaling. Our review also presents potential strategies for targeting NRF2 as an adjuvant therapy to overcome TMZ chemoresistance in glioblastoma. Specific molecular pathways, including MAPKs, GSK3β, βTRCP, PI3K, AKT, and GBP, that modulate NRF2 expression leading to TMZ resistance are discussed, along with the importance of identifying NRF2 modulators to reverse TMZ resistance and develop new therapeutic targets. Despite the significant progress in understanding the role of NRF2 in GBM, there are still unanswered questions regarding its regulation and downstream effects. Future research should focus on elucidating the precise mechanisms by which NRF2 mediates resistance to TMZ, and identifying potential novel targets for therapeutic intervention.

## 1. Introduction

Glioblastoma (GBM) is the most frequent and highly aggressive brain tumor subtype, comprising about 16% of all primary neoplasms that affect the brain and nervous system [1]. One of the characteristics of this type of tumor is its high level of heterogenicity, both at the cellular and molecular level. As a consequence, the identification of specific therapeutic targets is a great challenge in glioblastoma [2]. Thus, despite the advances that have been made to date, GBM remains one of the deadliest human cancers, with a median overall survival (OS) of approximately 15 months, a progression-free survival (PFS) of 7 to 10 months, and a five-year survival rate of only 5.1% [3]. The recommended treatment is usually based on a combination of maximal surgical resection, radiation therapy, and chemotherapy. After the surgery, the standard first-line postsurgical therapy consists on the use of temozolomide (TMZ), combined with regional fractionated radiotherapy, and is followed by additional cycles of adjuvant TMZ. The introduction of TMZ as a first-line treatment, since its Food and Drug Administration (FDA) approval in 2005, has improved patients’ quality of life and OS. However, cases of tumor resistance towards TMZ treatment are quite frequent [2,3,4].

Actually, it is a consensus in the scientific and clinical community that drug resistance is the main culprit in cancer treatment failure. Multiple mechanisms are involved in chemoresistance, for example, a lower rate of drug influx or higher drug efflux; the enhancement of drug inactivation; increased DNA repair capacity; and the inhibition of apoptotic signaling [5]. Remarkably, virtually all these mechanisms are modulated by Nuclear Factor Erythroid 2-related factor 2 (NRF2). In this review, we will discuss how typically NRF2 becomes constantly activated in GBM and the main mechanisms that are controlled by this transcription factor and that enable resistance to TMZ. Finally, it will be highlighted how NRF2 activity modulation in combination with this chemotherapy can improve the drug’s efficacy.

## 2. TMZ

TMZ is an imidazotetrazine, belonging to the class of alkylating chemotherapy drugs, and is administrated orally. It has high bioavailability and overall, it is well tolerated when compared to other chemotherapeutics, and induces mainly hematological side effects, such as lymphopenia, anemia, thrombocytopenia and neutropenia; these effects are observed in approximately 50% of patients [6,7,8]. This agent is a small molecule with a considerably high lipophilicity, which allows it to cross the blood–brain barrier (BBB) [9,10]. For these reasons, it is considered the most important systemic drug in the treatment of GBM. TMZ is a pro-drug and, at physiological pH, it is converted into the metabolite 5-(3-methyltriazen1-yl) imidazole-4-carboxamide (MTIC), whose hydrolysis leads to methyl-diazonium production, which can damage DNA [6]. TMZ produces a wide range of DNA damage, with the major product being guanine methylation at the N7 position (80–85%), followed by adenine methylation at the N3 position (8%) and guanine methylation at the O6 position (8%). N7-methylguanine (N7-meG) is not considered a toxic or mutagenic lesion but can lead to increased guanine clearance, which leads to the formation of abasic sites; these, in turn, exhibit mutagenic and toxic properties as they block DNA replication. N3-methyladenine (N3-meA) is a toxic and mutagenic lesion that induces replication block and A:T to T:A transversion. However, the effectiveness of TMZ as a DNA-damaging agent results primarily from the formation of the O6-methylguanine (O6-meG) lesion. The alkylation caused by TMZ ultimately induces single and double-strand damage to the DNA molecule, resulting in cell cycle arrest and apoptosis [11,12].

Despite the promising initial response, TMZ effectiveness is limited by tumor resistance, leading to treatment failure in the majority of patients (about 90%), who experience early recurrence. Once TMZ causes cytotoxicity through DNA damage, unsurprisingly, the main drivers of TMZ resistance in GBM are DNA repair pathways, especially via O6-methylguanine-DNA methyltransferase (MGMT) activity, which removes O6-meG lesions. Patients with MGMT promoter methylation, and therefore inactivated MGMT, showed greater survival rates after TMZ treatment. Furthermore, even patients who initially responded well to TMZ treatment, failed therapy due to acquired resistance [13].

However, while MGMT is a well-known determinant of TMZ susceptibility in GBM, other factors, such as enhanced antioxidant systems, also play a role in contributing to TMZ resistance. In fact, this alkylating agent triggers apoptosis through a considerable increase in reactive oxygen species (ROS) production, which is associated with DNA damage accumulation. Upon TMZ treatment, the cells induce DNA repair mechanisms, such as base excision repair (BER), mismatch repair (MMR), non-homologous end joining (NHEJ), and homologous repair (HR), and also induce the overexpression of antioxidant proteins, mainly through the NRF2 pathway. In light of its crucial protective role against TMZ-induced ROS, it is not surprising that NRF2 activity is increased in GBM cells following the TMZ treatment. Indeed, NRF2 nuclear hyperactivation was observed in recurrent GBM tumor tissues after TMZ treatment and was negatively correlated with time for tumor recurrence [14,15].

## 3. NRF2

NRF2 is a protein composed of 605 amino acids, which contains seven highly conserved NRF2-ECH domains, namely Neh1 to Neh7. This protein, encoded by the nuclear factor (erythroid-derived 2)-like 2 (*NFE2L2*) gene, is a transcription factor responsible for the expression of different antioxidant and xenobiotic detoxification genes, in response to the accumulation of ROS or electrophilic insults [16,17]. This protein is present in all types of cells, but its basal protein levels tend to be low under physiological conditions.

**Figure 1:**
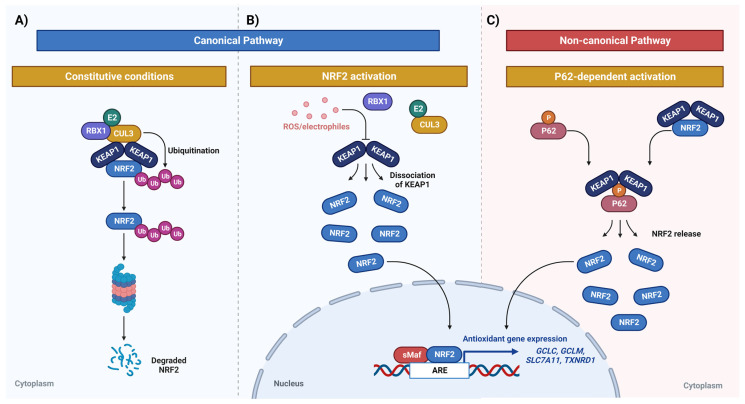
Canonical and non-canonical NRF2 pathways. (**A**) NRF2 proteasomal degradation under constitutive conditions. The ETGE and DLG motifs of NRF2 bind to the KEAP1 Kelch domains; this binding causes the ubiquitin ligase CUL3/RBX1 E2 to join the complex, in which CUL3 acts as a scaffold protein that binds to the BTB domain of KEAP1, allowing the formation of a complex with a ubiquitin-conjugating enzyme (E2). (**B**) Under oxidative and/or electrophilic stress, KEAP1 undergoes a conformational change, which interrupts the Kelch/DLG binding and leads to its detachment from NRF2, thus the degradation of NRF2 is interrupted and it translocates to the nucleus. (**C**) Non-canonical pathway of P62-mediated NRF2 activation. When autophagic flux is compromised and P62 accumulates, KEAP1 is sequestered by P62 and does not bind to NRF2, stopping its degradation. Created with BioRender.

The activation of NRF2, in its canonical pathway, is tightly regulated by Kelch-like ECH-associated protein 1 (KEAP1) via the ubiquitin proteasome system (UPS). More specifically, through the Kelch domain, KEAP1 interacts with the Neh2 domain of NRF2 in the cytoplasm, forming the NRF2/KEAP1 complex that becomes a substrate for CUL3, leading to the proteasomal degradation of NRF2 (Figure 1A). There are two binding motifs within the Neh2 domain, the low-affinity DLG motif and the high-affinity ETGE motif [18]. Upon oxidative stress exposure, the DLG motif dissociates from KEAP1 due to conformational changes, halting NRF2 degradation. NRF2 is then translocated to the nucleus [19], where it forms a heterodimer with Small Maf (sMAF), and binds to antioxidant response element (ARE) regions in the promoter of genes involved in redox balance, such as *GCLC, GCLM, SLC7A11* and *TXNRD1*, activating their transcription (Figure 1B). Of note, most somatic NRF2 mutations are found within these motifs [20]. NRF2 activation can also be regulated by the P62 protein, once it competes with NRF2 for interaction with KEAP1. KEAP1 has greater affinity with phosphorylated P62 than with NRF2, releasing NRF2 into the cytoplasm to bind to P62, culminating in the accumulation of cytoplasmic NRF2 and translocation to the nucleus (Figure 1C) [21].

Another method of NRF2 regulation occurs through the disruption of the interaction between KEAP1 and CUL3, which reduces NRF2 ubiquitination [18]. In addition to this process, NRF2 that escapes from KEAP1/CUL3 degradation can be captured by β-transducin repeat-containing protein (βTRCP) and Cullin 1, and then ubiquitinated and targeted for proteasomal degradation. This non-canonical signaling is mediated by the phosphorylation of serine residues in the Neh6 domain of NRF2 by glycogen synthase kinase 3 (GSK3), an important effector of the PI3K-AKT pathway [22].

Despite the fact that NRF2 activity is primarily regulated at the protein level, some molecular pathways, especially those activated by oncogenes, can regulate its transcription. In this sense, it has been shown that NRF2 can be transcriptionally activated by *KRAS*, *BRAF* and *MYC* in many types of cancer [23]. In addition, NRF2 can be controlled by epigenetic modification, for instance, the hypermethylation of CPG sites in the *KEAP1* promoter is frequent and leads to the downregulation of KEAP1 in several types of cancer [24,25,26,27]. In addition, there are reports indicating that some microRNAs (miRNA) can inhibit NRF2 expression, such as *miR-144* [28] and *miR-28* [29] and that some miRNAs, such as *miR-200a*, downregulate KEAP1 [30,31,32].

NRF2 is expressed in a wide range of cell types, but with different expression levels. For example, in the brain, neurons and endothelial cells have lower expression of NRF2 than monocytes and microglia. In addition, in the blood, monocytes and neutrophils show a high expression of the transcription factor [33].

### NRF2 in Cancer

**Figure 2:**
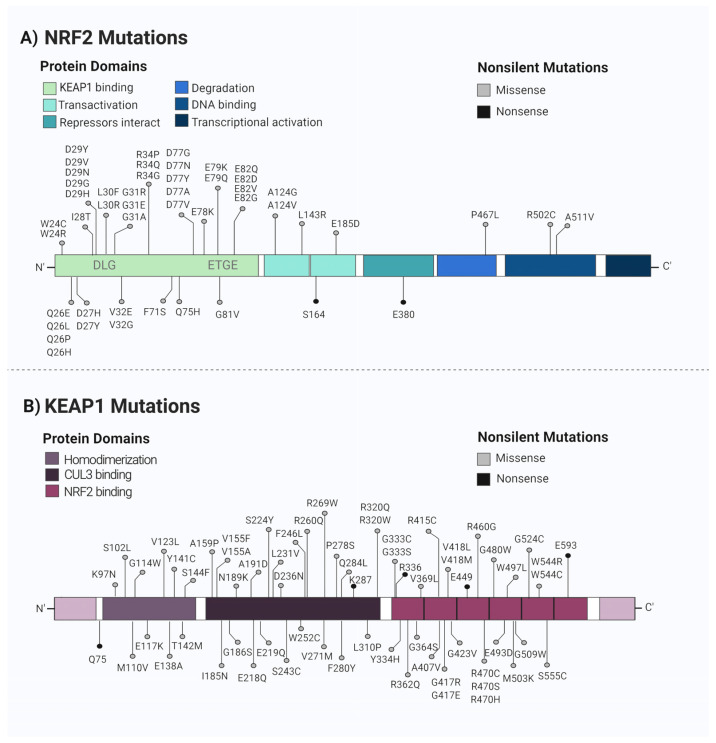
NRF2/KEAP1 mutations. (**A**) The somatic mutations in NRF2 are mostly localized in DLG and ETGE motifs, corresponding to the KEAP1 binding sites. These mutations lead to an increase in NRF2 activity. (**B**) As for KEAP1, the mutations are found throughout the entire gene, which leads to protein function loss, promoting, as a consequence, high levels of NRF2. All the reported mutations are listed in the COSMIC (Catalogue of Somatic Mutations in Cancer) database. Created with BioRender.

Although, for a long time, NRF2 was considered to be a tumor suppressor protein [34,35], in recent years, increasing evidence has suggested that NRF2 may be beneficial for the survival of not only normal cells, but also cancer cells, supporting the suggestion that NRF2 may contribute to tumor progression. Therefore, NRF2 has been considered to be a protumor in the advanced stages of cancer, since its hyperactivation protects cancer cells from oxidative stress, which increases antioxidant defense and leads to treatment resistance. Cancer-associated mutations that lead to NRF2 activation have been described in many types of tumors (Figure 2). These mutations are typically found in the motifs responsible for interaction with the KEAP1, which causes KEAP1/NRF2 binding impairment. Furthermore, KEAP1 loss-of-function mutations have been observed in a wide variety of carcinomas [23]. Importantly, the promoter region of KEAP1 has been reported to be hypermethylated in gliomas and is associated with poor prognosis [36].

As previously mentioned, oncogenes such as *MYC, KRAS*, and *BRAF* are capable of inducing NRF2 expression and activity, resulting in cytoprotective activity in the cell and decreased ROS levels. Consequently, NRF2 activity creates a more favorable intracellular environment for tumor cell survival and also protects against chemotherapeutic agents and radiotherapy [19,20]. However, it is still unknown whether NRF2 can promote tumor formation or whether the increase in NRF2 levels is a response to a more stressful situation induced by oncogenes [19].

It was demonstrated that NRF2 inhibition in prostate cancer cells resulted in a high sensitivity to anticancer drugs such as paclitaxel, cisplatin, and etoposide, leading these cells having a lower survival percentage and contributing to the suppression of growth both in vitro and in vivo [37,38,39]. In hepatocellular carcinoma cells, NRF2 can promote resistance to chemotherapy drugs, such as sorafenib, leading to a poor prognosis [40]. It was also shown that the suppression of NRF2 might increase the expression of other tumor suppressors, such as *miR-27b-3p*, which diminishes aggressive features related to the proliferation, migration, invasion, and epithelial-to-mesenchymal transition of esophageal squamous cell carcinoma (ESCC) [41].

Thus, while NRF2 may act in cancer prevention during the early stages of the disease, it can also contribute to tumor progression in advanced stages. This duality highlights the complexity of NRF2 participation in cancer. However, it is important to note that there is no difference in the mechanism in which NRF2 promotes cell protection, and that the only difference is in the scenario in which the cell is protected: for normal cells, NRF2 activation occurs in a controlled and transient manner, whereas for tumor cells, NRF2 is constitutively activated, conferring cytoprotection and leading to chemotherapy resistance [42]. Therefore, since NRF2 is a key molecular player involved in many fundamental cellular responses to chemotherapy, it has been explored over the past years as a promising therapeutic pathway in glioblastoma [43]. In this sense, a recent study from our group, utilizing CRISPR library screening, identified NRF2 as one of the main targets in the context of TMZ resistance, further emphasizing the importance of this pathway in glioblastoma [44].

## 4. NRF2 and TMZ Resistance

### 4.1. DNA Repair Mechanisms

Cancer chemoresistance is often found associated with the increased expression and/or activity of DNA repair proteins [45]. In this context, NRF2 plays a significant role in TMZ resistance by regulating the expression of multiple DNA repair genes, such as *MGMT* and *APE1*. As mentioned before, TMZ induces many different DNA lesions, with O6-meG being the most cytotoxic of them. MGMT repairs O6-meG by transferring the O6-methyl group from the modified guanine to the cysteine residue (Cys 145) in its active site in a stoichiometric irreversible reaction. Once it happens, there is a conformational change in the DNA binding domain of MGMT, leading the enzyme to the UPS degradation [46].

Around 50% of GBM tumors have *MGMT* epigenetically silenced by promoter hypermethylation at the *MGMT* locus, which is associated with a better prognosis in GBM patients [3]. Several drugs can inhibit the activity or expression of *MGMT* through different mechanisms, including O6-benzylguanine (O6-BG), O6-(4-bromothenyl) guanine (lomeguatrib), interferon-β (IFN-β) and levetiracetam (LEV) [46,47]. In terms of the mechanism of action, LEV can silence *MGMT* by increasing *HDAC1* transcription and recruiting the *HDAC1*/*mSin3A* corepressor complex to the p53-binding site in the promoter region of *MGMT* [47]. Importantly, all of the MGMT inhibitors are able to improve the overall survival of patients when combined with TMZ [46].

On the other hand, GBM tumors with high levels of *MGMT* and, consequently, increased DNA repair, commonly display TMZ resistance, and a strong TMZ chemo-resistant phenotype can be acquired with decreased *MGMT* promoter methylation over the course of tumor treatment, progression and recurrence; thus, the *MGMT* promoter methylation status is a crucial indicator of the patients’ outcome [3]. Interestingly, in glioma patients treated with radiotherapy and TMZ, it was found that the simultaneous methylation of *MGMT* and *KEAP1* promoters correlated with a lower risk of tumor progression, while patients showing methylated *MGMT* and unmethylated *KEAP1* had a worse prognosis; this is in agreement with the fact that NRF2 activation confers protection to cancer cells against the genotoxic damage caused by chemotherapy and radiotherapy [48,49]. Recently, it was reported that the *MGMT* promoter harbors two AREs, and that increased levels of MGMT were detected after transient and stable transfections with the NRF2 expression vector in MCF-7 and HT29 cells, suggesting that NRF2 is a regulator of MGMT [50].

In the absence of MGMT, the O6-MeG is mispaired with thymine (instead of cytosine) during replication, recruiting the MMR proteins that excise a portion of the newly synthesized DNA containing the thymine [3,46]. Then, with subsequent DNA synthesis, thymine is once again inserted opposite to the O6-MeG, resulting in futile cycles of MMR and the accumulation of DNA single-strand breaks (SSBs), which can eventually lead to replication fork collapse, cell cycle arrest and ultimately, apoptosis. For this reason, functional MMR is crucial for TMZ efficacy. In fact, mutations in essential components of MMR machinery, such as MLH1 and MSH6 in colon carcinoma cells, confer resistance to TMZ treatment [3,46]. Likewise, glioma cell lines, GBM xenografts and patient tumor samples bearing somatic mutations in *MSH2*, *MLH1* and *MSH6* exhibit TMZ tolerance after treatment, despite MGMT levels [3,46].

In addition to generating O6-meG DNA lesions, TMZ is also responsible for different DNA methylations, such as O3-MeA, N7-MeG and N3-MeA. Since N7-MeG and N3-MeA lesions are notably repaired by BER, many enzymes of this DNA repair pathway are involved in TMZ resistance in GBM, for instance, DNA glycosylase MPG and APE1, and DNA polymerase-β and PARP1 [3,46]. In this context, it was reported that TMZ cytotoxicity is further improved in combination with PARP inhibitors [3,46]. Furthermore, high APE1 expression levels are often found in human gliomas in association with TMZ resistance [3,46]. Regarding NRF2 and BER, it was reported that the NRF2 transcriptional target TRX1 can reduce residues of cysteine in the active site of APE1; additionally, PARP1 was found to be a coactivator of NRF2. Furthermore, 8-oxoguanine DNA glycosylase (*OGG1*) is regulated by NRF2 activity [49].

Moreover, NRF2 can also contribute to TMZ resistance once it regulates the expression of DNA repair genes containing AREs such as *BRCA1*, *53BP1*, *RAD51*, *RAD52*, *XRCC2, XRCC3, DMC1, RBBP8*, and *SHFM1*, although further studies are needed to confirm some of these genes as direct targets of the NRF2 pathway [49].

### 4.2. Drug Detoxification

There are more than 100 genes presenting AREs in their sequence and thereby being regulated by NRF2 [19,21]. NRF2 plays an important role in protecting against xenobiotic molecules, primarily through glutathione (GSH) induction, which can lead to chemoresistance [17,51]. GSH is a ubiquitous intracellular tripeptide (L-γ-glutamyl-L-cysteinyl-glycine) that can react to a variety of ROS and electrophilic agents; this is due to its sulfhydryl group that is present on the cysteine residue. Besides playing an important role in redox homeostasis, GSH is also intimately involved in the detoxification of various xenobiotic agents. In order to do this, glutathione-S-transferase (GST) enzymes use GSH as a co-factor in a conjugation reaction with a variety of electrophilic agents, including chemotherapy drugs; this results in a GSH–drug complex that can be exported out of the cell via efflux pumps, such as multiresistant-associated channels [52].

GSH synthesis occurs through two enzymatic processes: firstly, the glutamate cysteine ligase (GCL), formed by GCLM and GCLC, catalyzes the binding of glutamate to cysteine; and second, synthesized glutathione (GS) adds a glycine residue to the C-terminal of the γ-glutamylcysteine. The main limiting factor of GSH synthesis is the intracellular availability of cysteine, which is mainly controlled by the cystine/glutamate transporter system (xCT). Finally, GSH oxidation results in a glutathione disulfide (GSSG) that can be restored back to GSH via glutathione reductase (GR) activity [52].

**Figure 3:**
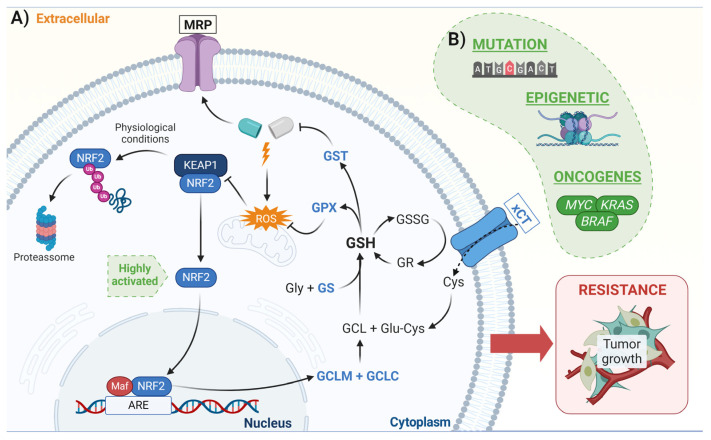
Drug detoxification mediated by NRF2 in cancer cells. The high activation of NRF2 promotes an increase in the GSH synthesis via the induction of GCLM and GCLC. (**A**) Once GSH is synthesized, the GST induces its binding to the chemotherapeutic drug and the GSH–drug conjugate is exported out of the cell through the multidrug resistance-associated proteins (MRPs) channel. In addition, GPX controls the ROS levels generated by the drug. These mechanisms lead to an increase in resistance to antitumor drugs in cancer cells and promote their growth and metastasis. (**B**) The green shape shows examples of mechanisms that induce NRF2 activation in cancer cells. Created with BioRender.

It has been widely demonstrated that NRF2 is the main mechanism involved in the regulation of GSH homeostasis. In this sense, NRF2 regulates the following: (i) GSH synthesis (by induction of GCLM and GCLC) [34]; (ii) cysteine exchange transporter [35]; (iii) GSH utilization (GPX2 and GST) [36]; and (iv) GSH recycling (GS) [53]. Many studies have identified a correlation between high levels of intracellular GSH and resistance to various antitumor drugs, including platinum-based drugs [54,55], anthracyclines [56], and alkylating agents [57]. In agreement with this, previous studies by our group showed that GBM cell lines that were counted to have high levels of GSH were resistant to the alkylating agent TMZ due to NRF2 overexpression [58,59]. Thus, the NRF2 pathway is an essential element in drug resistance through the induction of GCLM expression, leading to increased GSH content (Figure 3) [60].

### 4.3. Molecular Pathways That Modulate NRF2 Leading to TMZ Resistance

Besides KEAP1-dependent NRF2 regulatory pathways, as shown in Figure 1, there are also KEAP1-independent non-canonical regulatory pathways that lead to TMZ resistance, as shown below.

#### 4.3.1. MAPK Pathways

Mitogen-activated protein kinase (MAPK) comprises a family of evolutionarily related enzymes that play key roles in cellular signal transduction, controlling differentiation, survival, proliferation, and drug resistance [61]. There are several groups of MAPKs, of which the most common and best characterized are ERKs, JNKs and p38 kinases [62]. Among the proteins that are part of this pathway, RAS is involved in cellular signal transduction and has GTPase activity, in which it alternates between an active state, bound to GTP, and an inactive state, bound to GDP [61,63]. After RAS-GTP activation, it binds to RAF protein kinase, which exhibits specific kinase activity for serine/threonine residues, leading to the activation of the ERK/MAPK pathway. In a recent study, it was observed that in a U251 glioblastoma cell line treated with TMZ and NRF2 inhibitors, there was a decrease in the expression of RAS, followed by a decrease in MEK and ERK activation and a higher cell toxicity [64]. Another study showed that treating U87 and U251 with TMZ led to an increase in the transcriptional activity of NRF2 and the phosphorylation of P38 MAPK in a dose-dependent manner [65]. In addition, the pharmacological and genetic inhibition of p38 MAPK reduced the levels of many NRF2 targets, such as NQO1 and HO1, and increased the sensitivity of tumor cells to treatment with TMZ [66]. Thus, these data suggest that one of the ways in which NRF2 mediates tumor resistance to chemotherapy is through the activation of the MAPK pathway.

#### 4.3.2. GSK3β/βTRCP/NRF2 Pathway

GSK3 is a multifunctional serine/threonine kinase that plays diverse roles in the signaling pathways involved in proliferation and survival [67]. It is an enzyme that predominantly occurs in two isoforms: GSK3α and GSK3β. GSK3β activity is controlled through phosphorylation in tyrosine residues, and several kinases have already been identified as inhibitors of its activity, such as AKT and PKA [68]. One of the ways in which GSK3β acts as a player in cells’ resistance to chemotherapy drugs is through the regulation of NRF2 activity. In fact, it was first demonstrated that SCF/βTRCP promoted NRF2 degradation, in a way that was GSK3β-dependent and KEAP1-independent [69]. Recently, it was shown that this particular NRF2 degradation pathway is regulated by the CD147 protein and that it is involved in the resistance that glioma cells present to TMZ [70]. In this work, the authors showed that the CD147 is highly expressed in glioma cell lines, and that CD147 increases NRF2 expression by regulating the AKT/GSK3β pathway, inhibiting the βTRCP-mediated degradation of NRF2. Additionally, upon CD147 knockout, the authors observed a decrease in the expression of NRF2 and some of its targets, such as NQO1, HO1 and GCLC. Mechanistically, GSK3β phosphorylates NRF2 at Ser344 and Ser347 residues, enhancing the interaction between NRF2 and E3-ligase βTRCP, which results in increased ubiquitination and the consequent proteasomal degradation of NRF2 [69]. If GSK3β is inhibited by AKT or other enzymes, these serine residues are dephosphorylated and NRF2 is released from βTRCP, which allows nuclear translocation to take place. Therefore, the authors proposed that CD147 may regulate NRF2 expression through the AKT/GSK3β axis, as CD147 promotes NRF2 stability by suppressing NRF2 degradation in a GSK3β/βTRCP-dependent manner [70].

#### 4.3.3. PI3K/AKT Signaling Pathway

The PI3K/AKT pathway is a highly conserved pathway involved in cell survival, proliferation and metabolism, especially during cellular stress conditions [71]. The signaling cascade is initiated by the activation of receptor tyrosine kinases (RTKs), recruiting phosphatidylinositol 3-kinase (PI3K) to the membrane; this ultimately leads to the activation of signaling proteins, including the serine and threonine kinase AKT. Once active, AKT can regulate several downstream target genes related to glucose metabolism, apoptosis, and cell cycle progression [71]. A study observed that by secreting VEGF, hypoxic M2 macrophages were able to promote the activation of the PI3K/AKT/NRF2 pathway to enable TMZ resistance, as well as cancer aggressiveness and stemness in GBM cells [72].

In another work, NEDD4-1, an E3 ubiquitin ligase, was found to be upregulated in TMZ-resistant U87 and U251 GBM cell lines, and its elevated expression correlated with a worse prognosis in GBM patients [73]. NEDD4-1-mediated ubiquitination can modulate multiple substrates involved in several biological processes, including the tumor suppressor PTEN, the well-known negative regulator of the PI3K/AKT signaling pathway [74]. According to Chuang et al., the enhanced expression levels of NEDD4-1, triggered by the dysregulated expression of miRNAs (*miR-3129-5p* and *miR-199b-3p*), led to PTEN attenuation, which ultimately hyperactivated AKT signaling and induced NRF2/HO1 activity. Corroborating these findings, the inhibition of NEDD4-1 through indole-3-carbinol (I3C) treatment decreased tumorigenicity and increased the chemosensitivity of TMZ-resistant GBM cells to TMZ [73].

#### 4.3.4. cGAS-STING/GBP Signaling

Cyclic GMP-AMP synthase (cGAS) is a cytosolic DNA sensor that can recognize a broad spectrum of double-stranded DNA (dsDNA), including viral, bacterial, mitochondrial, micronuclei and retroelement origin DNA; this triggers an innate immune response that is mediated by STING [75,76]. In parallel, STING can induce a NFκB-driven inflammatory response through the activation of IκB kinase (IKK), which, in turn, could lead to angiogenesis and metastasis [75,76]. In recent years, the cGAS/STING pathway has received significant attention in the context of cancer. Like NRF2, cGAS/STING signaling also has an ambiguous role in cancers, being able to promote tumor development and metastasis, as well as an immune-suppressive tumor microenvironment if persistently activated [75,76]. The interconnection between NRF2 and STING was recently elucidated, indicating that NRF2 represses STING expression through post-transcriptional modification [77].

Similarly, GBP3, one of the seven members of the interferon-inducible large GTPase family in humans, plays an important role in the prevention of intracellular pathogenic infection. Furthermore, a report revealed that both GBP3 and STING exert functions outside of the immune system, reducing DNA damage induced by TMZ and apoptosis in GBM cells [76]. A pioneer study pointed out that the GBP3 is highly expressed in glioma, which induces ERK1/2 activation mediated by p62/SQSTM1 and the consequent glioma cell proliferation [78]. Subsequent studies demonstrated that GBP3 and STING expression can be stimulated after TMZ administration in GBM cells [76]. Moreover, the silencing of GBP3 and STING in glioma cells diminished the levels of NRF2 [76]. Therefore, the upregulation of these two proteins increases the expression of p62/SQSTM1, NRF2, and MGMT, which results in the enhanced repair of the DNA damage imposed by TMZ and, ultimately, by TMZ chemoresistance in GBM [76].

### 4.4. NRF2 Regulates Cell Death in TMZ-Resistant Cells

Even though the mechanisms of action and target pathways may differ for each chemotherapy drug, ultimately, the main objective of chemotherapy is to induce cell death [79,80,81,82]. It is known that NRF2 is an important regulator involved in many cell death processes. For instance, in the context of apoptosis, which is a process of programmed cell death that is essential for maintaining the development of living beings and is important for eliminating superfluous or defective cells, NRF2 is involved in the expression of antiapoptotic factors such as BCL2 and BCLxL and, consequently, in reducing cytochrome C release from mitochondria and the activation of caspase 3/7 [83]. Numerous studies have reported that TMZ-resistant GBM cells typically have high NRF2 expression levels, and that its silencing and/or pharmacological inhibition induce apoptosis in tumor cells, thus restoring chemosensitivity; this demonstrates the importance of NRF2 regulation in apoptotic cell death in TMZ-resistant GBM cells [84,85].

Ferroptosis is a distinct type of cell death that was described in 2012 by Dixon and colleagues [80]; it is classified as iron-dependent and is characterized by the loss of GPX4′s ability to repair lipoperoxidation, the presence of redox-active iron, and the oxidation of polyunsaturated fatty acid (PUFA)-containing phospholipids [86]. Several studies have shown that chemotherapy may induce ferroptosis in cancer cells [87]. In a recent study, it was described that TMZ triggers ferroptosis by regulating the DMT1 transporter, responsible for controlling iron levels in cells [88]. In fact, TMZ treatment in TG905 cells was able to increase iron levels, as well as increase the expression of DMT1 mRNA, which is directly related to ferroptosis.

Typically, NRF2 acts as a negative regulator of ferroptosis, since its inhibition sensitized head and neck cancer cell lines to ferroptosis [89]. Furthermore, in glioma cell lines, the overexpression of NRF2 led to ferroptosis resistance [90]. However, recent studies have shown that NRF2 expression can be involved in ferroptosis induction since NRF2 regulates some genes required in ferroptotic activation pathways, such as those involved in iron accumulation [91]. Accordingly, high NRF2 activity leads to an increase in the expression of HMOX1, a pro-ferroptotic protein that promotes iron accumulation [92,93]. In addition, in osteosarcoma cell lines, it has been described that the increase in NRF2 levels is related to a sensitization to ferroptosis, due to an increase in ABBC1 gene expression, thus counterbalancing the protective effect of NRF2 [94]. A study performed by our group revealed that high NRF2 expression leads to TMZ resistance, due to an increase in MRP1 levels. Importantly, the elevated amount of MRP1 was related to TMZ-resistant GBM cells, but sensitized these cells to treatment with ferroptosis inducers [95].

Autophagy is a catabolic process that is activated in stressful situations and that degrades abnormal proteins, other biomolecules (such as lipids and nucleic acids), organelles and intracellular pathogens inside lysosomes [96], providing metabolic precursors in nutrient deprivation conditions [97]. In cancer cells, depending on the tumor stage, autophagy can play different roles, being anti or pro-tumor [96]. If the tumor has already been formed, autophagy is strictly associated with survival and resistance to chemotherapy. Therefore, the use of autophagy inhibitors combined with standard chemotherapies has been proposed [98,99]. However, autophagy is also associated with cell death induction, which may explain why so many clinical trials using autophagy inhibitors failed to improve overall survival for cancer patients.

P62 is a well-established protein involved in the execution of autophagy, delivering ubiquitinated proteins and organelles to autophagic degradation [100,101]. Besides that, p62 also promotes the degradation of KEAP1, indirectly controlling the levels of NRF2 [21,102], and thus increasing its bioavailability [21,103]. The augmented levels of p62 and also NRF2 can contribute to tumorigenesis and tumor progression, in addition to chemotherapy resistance [104,105]. The relationship between TMZ, NRF2 levels and autophagy has also been described in gliomas. According to Zhou and colleagues, the knockdown of NRF2 increases autophagy induced by TMZ in glioma cells [106]. In U251 siNrf2 cells, the levels of LC3-II, autophagic vacuoles and acidic vesicular organelles were measured, showing an increase in the levels of these autophagy markers [106]. These findings provided evidence that NRF2 also modulates the autophagic pathway [106].

## 5. Compounds That Modulate NRF2

Drug resistance has been consistently associated with high levels of NRF2 expression in glioma tissues, and as a consequence, NRF2 has become a potential new target for GBM treatment [107]. Indeed, TMZ treatment, combined with irradiation, increased the levels of NRF2 and its targets, preventing cell death [108]. Thus, several studies have focused on identifying compounds that modulate NRF2 activity or its pathway in brain tumors. Here, we summarize recent studies on potential NRF2 modulators that could indicate favorable strategies for glioma treatment, and guide the discovery and development of novel NRF2 modulators (Table 1).

Vaproic acid (VPA) and melatonin (MEL) act as NRF2 inhibitors that, in combination with TMZ, lead to the sensitization of glioma-resistant cells. These compounds impair the phosphorylation of the IGFR/AKT/mTOR signaling pathway, blocking the NRF2/ARE signaling pathway [84]. Retinoic acid (RA) is a chemotherapeutic drug that suppresses cell growth by inducing cell death via apoptosis [125]. It was observed that RA increased the inhibitory effects of TMZ on cell proliferation, cell cycle arrest, and induction of apoptosis and autophagy. Western blot and qPCR analyses confirmed that these cytotoxic effects of TMZ + RA occur via the downregulation of the KEAP1/NRF2/ARE signaling pathway [116].

An active natural bioflavonoid known as chrysin (5,7-dihydroxyflavone) has an anti-cancer effect in human GBM cells in a time- and dose-dependent manner. This natural compound hinders the proliferation, migration, and invasion capacity of cells via NRF2 inhibition and a decrease in a subfamily of MAPKs, ERK1/2. Interestingly, in vitro assays revealed that the U87 cell line was highly susceptible to chrysin, and in vivo tumorigenicity experiments on this same cell line demonstrated that chrysin inhibited tumor growth [123].

LQB-118 is a promising synthetic compound for cancer treatment because it presents low toxicity, it can be orally administrated and it can pass through the BBB. In GBM cells, LQB-118 has shown antitumor activity through the induction of apoptosis, and a reduction in cell migration in a three-dimensional culture [4]. Studies have indicated that LQB-118 concurrently inhibits ERK, AKT, and P38 activation, followed by a reduction in NRF2 levels, which promotes a synergistic effect when combined with ionizing radiation and chemotherapy [4]. Likewise, FTY720, a synthetic compound from *Isaria sinclairii*, enhanced the sensitivity of U251 and U87 cell lines towards TMZ through the inhibition of NRF2 and its targets HO1 and NQO1 [85,126]. Notably, the FDA has approved the use of FTY720 in the treatment of relapsing–remitting multiple sclerosis (RRMS) [127].

Recently, it was identified that a novel small-molecule drug, termed CET-CH-6, has a potential effect in NRF2 inhibition. Researchers have developed a biosensor that expresses the (NQO1-FLuc) reporter cell line, which measures the transcriptional activation of NRF2; they found two compounds that are able to inhibit NRF2 activity around 4 to 5-fold. Indeed, treatment with CET-CH-6 synergistically enhances the therapeutic effects of TMZ and doxorubicin in P53-mutant glioma cell lines by inhibiting NRF2, both in vitro and in vivo [122].

The isoflavonoid brazilin has also been demonstrated to have anticancer effects on several types of tumors [109]. In GBM, brazilin treatment inhibited cell proliferation and induced cell cycle arrest and apoptosis [128]. In breast cancer cells, brazilin inhibited hemin-induced HO1 expression through the inactivation of the JNK/NRF2 pathway [110]. Furthermore, brazilin demonstrated a synergistic effect with chemotherapy, indicating an interesting mechanism of cytotoxicity via NRF2 modulation; this needs to be further investigated in glioma cells [110].

Corilagin has been associated with several pharmacological activities, including antitumor, antioxidant, and anti-inflammatory activities [129]. Increasing evidence has demonstrated that corilagin can cross the BBB, indicating its potential use for GBM treatment [129]. Thus, Liu and coworkers tested the effect of this compound in glioma cells, and showed that corilagin can decrease NRF2 protein levels, which leads to cell death via autophagy [111]. Compounds containing the imidazo [1,2-a] pyridines (IPs) heterocycle system have also played an important role in cancer chemotherapy, by inducing cell cycle arrest and cell death [130]. Similarly, synthetic organoselenium compounds have been widely reported in antitumor studies because of their ability to induce ROS production, DNA damage and autophagy in tumor cells [131]. Consequently, the molecular hybridization of these two compounds (IP-Se-06) decreased the levels of GSH and NRF2 in glioma cells, indicating a potentiated cytotoxic effect by altering the intracellular redox state [114]. Additionally, it was reported that IP-Se-06 can penetrate the BBB [114].

Recently, a compound named triptolide was reported to be a potent NRF2 inhibitor. Triptolide downregulated NRF2 and its targets, GCLM, GCLC, and SLC7A11, which compromised GSH synthesis and induced oxidative damage accumulation [124]. Triptolide exhibited selective cytotoxicity in IDH1-mutated glioma cells both in vitro and in vivo, indicating a promising therapeutic alternative for IDH1-mutated malignancies [124]. Another compound that deserves attention is shikonin, a naphthoquinone compound extracted from the roots of *Lithospermum erythrorhizon*. It was demonstrated that shikonin induces apoptosis in human glioma cell lines, including U87, Hs683, and M059K cells [132]. In addition to that, Yang and coworkers showed that shikonin inhibited the nuclear translocation of NRF2 in glioma cells (U87) by increasing intracellular ROS levels [118]. Therefore, shikonin represents a possible new chemotherapeutic strategy against human gliomas [118]. However, due to the low solubility and bioavailability of this compound and its short retention time [117], its anticancer potential becomes limited, mainly because of the BBB, which decreases the targeting capacity of shikonin in the brain. In this context, to optimize its anti-glioma effect, Li et al. demonstrated that shikonin encapsulated in nanoparticles displayed a greater potential to cross the BBB [117].

Fullerol is a chemical compound that acts as a ROS scavenger. In U87 cells infected with Zika virus (ZIKV), fullerol restored the transcriptional activity of NRF2 that was blocked by the infection [112]. This indicates that this compound plays an interesting role in NRF2 modulation under stressful conditions, but the mechanisms by which fullerol normalizes the NRF2 pathway remain unclear [112].

Cannabidiol (CBD) is a non-toxic and non-psychoactive cannabinoid that has been studied for its antitumor properties. Singer et al. observed that CBD treatment exhibited significant antitumor effects in GSCs by increasing ROS in vitro and in vivo; however, a subset of GSCs activated the NRF2 antioxidant response, which was inhibited by the synergistic effect of CBD and SLC7A11 inhibitor treatment [120]. It remains unclear as to whether CBD directly regulates NRF2; however, one study has indicated that CBD-based compounds, in combination with other small molecules, have the potential to inhibit GBM progression [120]. Furthermore, CBD has been approved by the FDA for the treatment of severe seizure disorders and it is important to note that some of these compounds have been studied in clinical trials [121]. For example, FTY720 had been tested to reduce the side effects generated by radiation and TMZ treatment in newly diagnosed high-grade glioma [85]. However, the drug did not pass phase I clinical trials due to drug-induced lymphopenia, increasing the incidence of infections (NCT02490930).

Currently, all-trans-retinoic acid (ATRA) is in phase II clinical trials in combination with a PD1 inhibitor (Retifanlimab) in patients with recurrent IDH-mutant gliomas. The study will be completed in 2026 and proposes that the combination could stimulate an anti-tumor immune response in surgical patients or patients that have not responded to treatment with TMZ and/or other alkylating therapy (NCT05345002). Valproic acid is also consistently present in clinical trials in glioma patients and it is being tested in combination therapy with other drugs, including TMZ and radiotherapy (NCT03243461/NCT00302159).

On the other hand, the pharmaceutical compound SFX-01, generated by the complexation of the active compound sulforaphane (SFN) with α-cyclodextrin, has been reported to have a antiproliferative capacity in GBM models in vitro and in vivo through various cellular mechanisms, including DSBs induction caused by ROS accumulation, reduced DNA repair, autophagy and, ultimately, caspase-dependent apoptosis [133]. SFN is known to be the most potent natural activator of the NRF2 signaling pathway. In fact, it was observed that the SFX-01 treatment induced NRF2 expression in glioma cells; however, the NRF2 pathway was not completely activated, since the levels of antioxidant response enzymes regulated by NRF2, such as SOD1 and SOD2, were found unchanged after treatment, which was indicated by the persistence of elevated ROS levels [133]. The drug was already tested in phase II clinical trials in ER-positive metastatic breast cancer patients (NCT02970682), and promising results were demonstrated regarding drug safety, tolerance and efficacy [133]. Moreover, SFX-01 is able to cross the BBB and, thus, presents a good potential for GBM treatment.

## 6. Conclusions

The key point in the TMZ resistance issue in GBM is ultimately associated with a lack of knowledge about the fundamental mechanisms that lead to treatment failure. Due to GBM heterogeneous characteristics, TMZ resistance, in general, is not based on a single mechanism, but rather relies on a set of them. Once NRF2 modulates several cellular responses against TMZ (such as antioxidant and detoxification processes; DNA repair and cell death regulation), it figures as an interesting therapeutic target to be explored. Furthermore, a large number of studies have associated NRF2 overexpression with TMZ resistance and with a more malignant GBM phenotype. Importantly, as different molecular pathways—such as the MAPKs, GSK3β, βTRCP, PI3K, AKT pathway—regulate NRF2 expression in cancer cells, future investigations are needed to elucidate the main mechanism involved in NRF2 availability in GBM. Finally, the development of powerful and specific NRF2 inhibitors is of the utmost importance in order to overcome TMZ resistance, and this highlight new potential therapeutic targets for GBM by modulating NRF2.

## Figures and Tables

**Table 1:** Anti-tumor compounds that regulate NRF2 signaling in glioma.

Compound	Dose	In Vitro/In Vivo	Cell Line or Animal Model	Mechanism	BBB-Crossing Capacity	Ref.
Brazilin	7.5–10 µM	In vitro	U87, MCF7 (Breast cancer)	Downregulation of HO-1, and JNK/NRF2 (in MCF7 cells)	N/A	[109,110]
Corilagin	25–100 µg/mL	In vitro	U251	NRF2 downregulation	Yes	[111]
Fingolimod (FTY720)	3.75–15 µM	In vitro	U251 and U87	Downregulation of NRF2, HO-1, and NQO-1	N/A	[85]
Fullerol	6.25 nM	In vitro	U87	NRF2 pathway induction under stressful conditions	N/A	[112]
Hinokitiol	25–100 µM	In vitro	U87 and T98G	NRF2 downregulation	N/A	[113]
IP-Se-06	1 µM	In vitro	A172, HT-22	NRF2 and GSH downregulation	Yes	[114]
LQB-118	3–12 µM	In vitro	U251, T98G, A172 and LAPC4, PC3 and LNCaP (Prostate cancer).	Inhibition of ERK, Akt and p38 activation, and reduction of NRF2	Yes	[4,115]
Melatonin (MEL)	0.1–2 mM	In vitro	U251	Inhibition of the NRF2-ARE signaling pathway	N/A	[84]
All trans-Retinoic acid (ATRA)	4 –20µM	In vitro	U251	Downregulation of KEAP1/NRF2/ARE	N/A	[116]
Shikonin	2 μM or 8 μM	In vitro	U87	NRF2 downregulation	Yes (encapsulated in nanoparticles)	[117,118]
Valproic acid (VPA)	0.5–5 mM	In vitro	U251	Inhibition of the NRF2-ARE signaling pathway	N/A	[84]
Baicalin LNCs	8–18 µg/mL	In vitro/in vivo	U87	NRF2 and HO-1 downregulation	Yes	[119]
Canabidiol (CBD)	In vitro: 0.5–2 µM In vivo: 15 mg/kg	In vitro/in vivo	U251, GSC lines 387 and 3832	N/A	Yes	[120,121]
CET-CH-6	In vitro: 2.5–30 µM In vivo: 2.5 mg/kg body weight	In vitro/in vivo	U87, LN308, NSG mice	NRF2 inhibition	N/A	[122]
Chrysin (5,7 dihydroxyflavone)	In vitro: 10–120 µM In vivo: 40–80 mg/kg	In vitro/in vivo	T98G, U251, U87	NRF2 pathway downregulation via inhibition of ERK signaling	N/A	[123]
Triptolide	In vitro: 30 nM In vivo: 0.5 mg/kg	In vitro/in vivo	U251, GSC827, GSC9, TB096, TS60	NRF2 and GSH downregulation	N/A	[124]

## Data Availability

No new data were created or analyzed in this study. Data sharing is not applicable to this article.
